# Re: Joris G. Heetman, Lieke Wever, Leonor J. Paulino Pereira, Roderick C.N. van den Bergh. Clinically Significant Prostate Cancer Diagnosis Without Histological Proof: A Possibility in the Prostate-specific Membrane Antigen Era? Eur Urol Open Sci 2022;44:30–2: *Avoiding Prior Biopsy Before Radical Prostatectomy: Innovation or Sacrilege?*

**DOI:** 10.1016/j.euros.2022.09.023

**Published:** 2022-11-17

**Authors:** Prasanna Ram

**Affiliations:** Department of Urology, All India Institute of Medical Sciences-Bhubaneswar, Bhubaneswar, India

I read the recent article by Heetman et al [1] in *European Urology Open Science* with great interest. In their retrospective study, the authors analyzed prostate-specific membrane antigen (PSMA) positron emission tomography/computed tomography (PET/CT) scans of 451 patients over 2 yr and found that all 74 patients with a PSMA standardized uptake value (SUVmax) value of ≥16 had clinically significant prostate cancer (csPC). Of the 185 patients who had a Prostate Imaging-Reporting and Data System (PI-RADS) score of ≥4 and SUVmax of ≥8, 98% had csPC. Studies suggest that lesions with a high SUVmax on PSMA PET/CT and a PI-RADS score of ≥4 on magnetic resonance imaging (MRI) have a high probability of csPC [2]. The authors referred to the PRIMARY trial and suggested a PRIMARY score for local activity on PSMA PET/CT [3]. They concluded that addition of PSM-based PET/CT could help in the diagnosis of csPC without the need for prostate biopsy, and that direct therapy without biopsy confirmation of cancer might be possible for a highly select group of individuals.

This concept, albeit provocative and intriguing, is still going to be met with a certain degree of skepticism. Unsurprisingly, no patient would opt for a radical procedure just to side-step the morbidity of a prostate biopsy.

Growing interest in the use of novel imaging techniques to detect prostate cancer has seen increasing utilization of modalities such as multiparametric MRI (mpMRI) and PSMA PET/CT. However, studies have proven that mpMRI misses approximately 17% of significant cancers and has a low positive predictive value [4]. Meissner et al [5] published a case series that suggested that this pathway is an option for patients refusing prostate biopsies.

One has to keep in mind that both mpMRI and PSMA PET/CT are observer-dependent, and the accuracy of both imaging modalities is not close to 100%. Until the observer variable is removed, one may need to be wary of the inevitable delay in implementation in clinical practice.

Many may feel uncomfortable about the concept of radical treatment without histologic confirmation. Traditionalists could argue that one should never proceed with radical surgery without tissue diagnosis. Here it is important to note that in current urologic practice, there are many examples of standard-of-care surgical procedures without prior biopsy, including orchiectomy, adrenalectomy, and nephrectomy. That being said, obtaining a confirmatory tissue diagnosis before surgery offers additional benefits in counseling patients regarding management options and important details for individualizing surgical or radiation treatment.

Finally, the study by Heetman et al [1] raises important and interesting ethical questions. How were these patients counseled? What if no cancer was present on final pathology? As in the above study, overestimation was a possibility. One has to note that a biopsy would provide detail regarding tumor characteristics and any need for DNA testing when planning radical or focal surgery. One should remain open-minded about the potential of new horizons while at the same time be steadfast in the scrutiny and evaluation of these ideas before adoption.

Lastly, I would like to congratulate the authors on their endeavors. This study could provide a pathway for future research.

  ***Conflicts of interest***: The author has nothing to disclose.
